# Prevalence and Molecular Epidemiology of *Staphylococcus aureus* among Residents of Seven Nursing Homes in Shanghai

**DOI:** 10.1371/journal.pone.0137593

**Published:** 2015-09-04

**Authors:** Ji Zhang, Fei-Fei Gu, Sheng-Yuan Zhao, Shu-Zhen Xiao, Yan-Chun Wang, Xiao-Kui Guo, Yu-Xing Ni, Li-Zhong Han

**Affiliations:** 1 Department of Clinical Laboratory, Shanghai People's Hospital of Putuo District, Shanghai, China; 2 Department of Clinical Microbiology, Ruijin Hospital, Shanghai Jiao Tong University School of Medicine, Shanghai, China; 3 Department of Microbiology and Parasitology, Shanghai Jiao Tong University School of Medicine, Shanghai, China; Ross University School of Veterinary Medicine, SAINT KITTS AND NEVIS

## Abstract

**Background:**

Residents in nursing homes (NHs) always represent potential reservoirs for *Staphylococcus aureus* and methicillin-resistant *S*. *aureus* (MRSA). To our knowledge, there is no epidemiological information up till now that describes the prevalence and molecular characteristics of *S*. *aureus* in nursing home residents in Shanghai, China.

**Methods:**

Four hundred and ninety-one unique residents from 7 NHs were enrolled in this study. Specimens were collected among these residents including 491 nasal swabs, 487 axillary swabs and 119 skin swabs. *S*. *aureus* isolated and identified from the swabs was characterized according to antimicrobial susceptibility profiling, toxin gene prevalence, and multilocus sequence typing (MLST), *spa* and SCC*mec* typing.

**Results:**

Among the 491 residents screened, *S*. *aureus* was isolated in 109 residents from 90 nasal swabs (90/491, 18.3%), 29 axillary swabs (29/487, 6.0%), and 22 skin swabs (22/119, 18.5%). Sixty-eight MRSA isolates were detected in 52 residents from 41 nasal carriers, 15 axillary carriers and 12 skin carriers. The overall prevalence rate of *S*. *aureus* and MRSA colonization was 22.2% and 10.6% respectively. Ten residents presented *S*. *aureus* in all three sample types and 12 residents presented *S*. *aureus* in two of the three sample types collected. Molecular analysis revealed CC1 (29.1%) to be the dominant clone in this study, followed by CC398 (19.9%), CC188 (13.5%) and CC5 (12.8%). The most common *spa* type was t127 (22.0%), followed by t14383 (12.8%) and t002 (10.6%).

**Conclusions:**

A high prevalence of *S*. *aureus* and MRSA colonization was revealed in nursing home residents in Shanghai. CC1 was the most common clonal complex and t127 was the most common *spa* type among NH residents. The data provides an important baseline for future surveillance of *S*. *aureus* in NHs in Shanghai and other highly urbanized regions in China. Implementation of infection control strategies must be given high priority in NHs to fight such high prevalence of both MRSA and methicillin-susceptible *S*. *aureus* (MSSA).

## Introduction

*Staphylococcus aureus* is a major pathogen which causes a wide range of human infectious diseases. In the past several years, methicillin-resistant *S*. *aureus* (MRSA) has increased in incidence in many parts of the world as agents of nosocomial infections [1]. *S*. *aureus* and MRSA are major causes of morbidity and mortality worldwide and have leaded significant social and economic consequences [2]. In addition, MRSA colonization rates are typically higher in elder people and the rate of *S*. *aureus* colonization also increases with advancing age [3]. As such, elderly people who reside in nursing homes (NHs) for long-term care represent an important reservoir for *S*. *aureus* and residence in a NH is a well-established risk factor for *S*. *aureus* carriage and infection[4]. As consequence, MRSA colonization in NH residents is also associated with higher mortality rates [5]. *S*. *aureus* is often carried on the skin surface and mucosa of healthy individuals without causing any harm, but in some patients *S*. *aureus* can cause severe infections and bacteremia leading to high mortality. A study has shown that there might be a substantial increase in incidence of *S*. *aureus* bacteremia over a 7-year period in NHs due almost exclusively to an increased occurrence of MRSA colonization [6].

Various studies have demonstrated high colonization rates of MRSA in residents in NHs with range from 4.7% to 23.3% in Europe[3, 7], 22% in the United States [2], and the prevalence rate of *S*. *aureus* in Sweden was up to 48%[8]. One study about MRSA epidemiology among residents in skilled nursing and intermediate care facilities in Hawaii has observed the proportions of MRSA which increased from 35.0% in 2000 to 58.6% in 2005 (P<0.001) during the 6-year period[9]. NHs has indeed represented a unique and important *S*. *aureus* reservoir. Furthermore, *S*. *aureus* can also be transmitted back into hospitals and the community from NHs. One recent modeling study has demonstrated that the addition of NHs substantially has changed the dynamics of MRSA transmission [10]. Another study concluded that one important risk factor independently associated with MRSA colonization in patients admitted from NHs was occurrence of previous hospital admissions[11]. As of 2014, Shanghai had about 600 certified NHs with over 96,000 residents on any given day. To our knowledge, there is still no information characterizing the *S*. *aureus* carried by NH residents in Shanghai.

The aims of our study were to determine the prevalence of *S*. *aureus* carriage in NH residents in Shanghai, and to characterize the molecular epidemiology of *S*. *aureus* from these NHs. Predictors thereof will help form infection control strategies for minimizing *S*. *aureus* prevalence and transmission in NHs, thus reducing the social and economic impact of NH-associated *S*. *aureus* nearby hospitals and the local community.

## Materials and Methods

### Study design

This was an active epidemiological surveillance study of *S*. *aureus* colonization in specimens sourced from nasal, axillary and skin samples from residents in 7 NHs in Shanghai, and the study was approved by the Ruijin Hospital Ethics Committee (Shanghai Jiao Tong University School of Medicine). This research is a descriptive and pilot study which focuses on the molecular epidemiology of *S*. *aureus* isolated from seven NHs in Shanghai. Written informed consent was obtained for each resident enrolled in the study or from a legal representative for residents with a cognitive disorder.

### Resident information and isolate collection

The median age of the study population was 82 years (range: 47–101 years) and the sex distribution (male/female) was 30.8%/69.2% and the median length of stay in the NHs was 10 months (range: 1–194 months). Participating NHs ranged in bed size from 45 to 190 beds and there was a grand total of 560 beds.

491 residents from 7 NHs were enrolled in this study, and 491 nasal swabs, 487 axillary swabs and 119 skin swabs were collected from these residents between March 8 and April 26, 2014. All swabs were cultivated on blood agar plates, incubated over night, and suspected *S*. *aureus* colonies were identified with DNase, mannitol fermentation and tube coagulase tests. *S*. *aureus* strains were stored at-80°C in nutrient broth containing 30% glycerol.

### Antimicrobial susceptibility testing

Antimicrobial susceptibility testing was performed following the Clinical and Laboratory Standards Institute guidelines[12] using the disk diffusion method and the following antibiotics: penicillin (10 units), cefoxitin (30 μg), gentamicin (10 μg), kanamycin (30 μg), tobramycin (10 μg), erythromycin (15 μg), tetracycline (30 μg), teicoplanin (30 μg), minocycline (30 μg), ciprofloxacin (5 μg), clindamycin (2 μg), sulfamethoxazole-trimethoprim (25 μg), chloramphenicol (30 μg), rifampicin (5 μg), quinupristin-dalfopristin (15 μg), linezolid (30 μg), fusidic acid (10 μg) and mupirocin (5 μg and 200 μg). The minimum inhibitory concentration (MIC) of vancomycin was detected by E-test. The penicillin disk diffusion zone edge test was performed for β-lactamase detection, and inducible clindamycin resistance was determined by the D-test as previously described[13]. *S*. *aureus* ATCC25923 and ATCC29213 were used as quality controls for the disk diffusion test and MIC detection respectively.

### Molecular typing

For confirming presence of MRSA, *mec*A detection was performed on all *S*. *aureus* collected. SCC*mec* types of MRSA were determined by a method previously described[14]. Molecular typing including multilocus sequence typing (MLST) and *spa* typing were performed on all *S*. *aureus* as previously described[15].

### Toxin gene detection

A variety of clinically significant toxin genes were detected using polymerase chain reaction (PCR)[13], including *lukS/F-PV* (encoding Panton-Valentine leukocidin); *tst* (encoding toxic shock syndrome toxin 1); *eta* and *etb* (encoding exfoliative toxin A and B); *sea*-*see* and *seg*-*sej* (encoding staphylococcal enterotoxins SEA-SEE and SEG-SEJ); and *sasX* (encoded in a mobile genetic element) which also acts as a virulence determinant and plays a key role in MRSA colonization and pathogenesis as reported by Li *et al*.[16].

### Statistical analysis

The chi-square or Fisher’s exact test were used where appropriate using the software package SAS 8.2 (SAS Institute Inc., Cary, NC, USA). A two-sided P value of < 0.05 was considered statistically significant.

## Results

### Prevalence of *S*. *aureus* and MRSA colonization

A total of 491 residents from 7 nursing homes were screened between March 8 and April 26, 2014. Nasal swabs were collected from all the residents (n = 491), axillary swabs were collected from 487 residents, and skin swabs were collected from 119 residents. *S*. *aureus* was isolated in 109 of the 491 residents screened, corresponding to an overall prevalence rate of 22.2%. *S*. *aureus* isolates from 87 residents were detected from one sample only (nasal, n = 71; axillary, n = 8; skin, n = 8). *S*. *aureus* isolates from 12 residents were detected from two of the three samples (nasal & axillary, n = 8; nasal & skin, n = 1; axillary & skin, n = 3), and *S*. *aureus* isolates from 10 residents were found detected in all three sample types. In total, *S*. *aureus* was isolated from 90 total nasal swabs (90/491, 18.3%), 29 axillary swabs (29/487, 6.0%), and 22 skin swabs (22/119, 18.5%).

A total of 68 MRSA isolates were found in 52 NH residents, including 41 MRSA isolated from nasal carriers (41/91, 45.6%; 41/491, 8.4%); 15 MRSA isolated from axillary carriers (15/29, 51.7%; 15/487, 3.1%) and 12 MRSA isolated from skin carriers (12/22, 54.5%; 12/119, 10.1%). The prevalence rate of MRSA colonization was 10.6%. All 68 MRSA isolates in this study were both *mecA* positive and cefoxitin resistant, and *mecC* gene was not found positive among 141 *S*. *aureus* isolates.

### Antimicrobial resistance

We did not discover any isolates resistant to vancomycin, teicoplanin, rifampicin, quinupristin-dalfopristin and linezolid. The resistance rates of other antibiotics tested for the *S*. *aureus* strains are presented in Table 1. Fifteen isolates (10 from nasal carriers; 2 from axillary carriers; 3 from skin carriers) were susceptible to penicillin with β-lactamase negative, and 41 isolates (25 from nasal carriers; 9 from axillary carriers; 7 from skin carriers) were inducible resistant to clindamycin based on D-test results.

**Table 1:** Antibiotic resistance rates of *S*. *aureus* isolated from residents in 7 NHs in Shanghai.

Antibiotic	Resistance rates (%)		
	Nasal (n = 90)	Axillary (n = 29)	Skin (n = 22)
Penicillin	88.9	93.1	86.4
Oxacillin	45.6	51.7	54.5
Gentamicin	3.3	24.1	13.6
Kanamycin	35.6	48.3	36.4
Tobramycin	36.7	37.9	40.9
Erythromycin	43.3	41.4	31.8
Tetracycline	30	24.1	4.5
Minocycline	7.8	20.7	0
Ciprofloxacin	33.3	44.8	27.3
Clindamycin^a^	21.1	24.1	0
Sulfamethoxazole-trimethoprim	1.1	0	0
Chloramphenicol	2.2	3.4	0
Fusidic acid	3.3	0	0
Mupirocin (5μg)	7.8	17.2	27.3
Mupirocin (200μg)	7.8	17.2	27.3

^a^ 41 isolates (25 from nasal carriers; 9 from axillary carriers; 7 from skin carriers) were D-test positive, indicating inducible clindamycin resistance.

### Virulence factors

The *sec* was the most frequently found among the toxin genes we screened for, occurring in 28 isolates (31.1%) from nasal carriers and 9 isolates (31.0%) from axillary carriers, while *sec* and *seh* were most frequently found in skin carriers (both 6/22, 27.3%). *etb*, *see* and *sasX* were not detected among all *S*. *aureus* isolates, and *lukS/F-PV* was found in only two nasal carriers and one axillary carrier, one of which belonging to ST88-t4333, and the other two isolates from the same resident were belonging to ST59-SCC*mec*V-t437. Presented in Table 2, we have not found any significant differences in prevalence among the toxin genes except *seg* (P = 0.0241) within the three groups.

**Table 2:** Prevalence of toxin genes among *S*. *aureus* isolated from residents in 7 NHs in Shanghai.

	Nasal (n = 90) n (%)	Axillary (n = 29) n (%)	Skin (n = 22) n (%)	P-value
*lukS/F-PV*	2 (2.2)	1 (3.4)	0	0.7431
*tst*	8 (8.9)	1 (3.4)	2 (9.1)	0.6325
*eta*	3 (3.3)	2 (6.9)	1 (4.5)	0.7036
*etb*	0	0	0	-
*sea*	23 (25.6)	7 (24.1)	5 (22.7)	0.9584
*seb*	17 (18.9)	6 (20.7)	1 (4.5)	0.2275
*sec*	28 (31.1)	9 (31.0)	6 (27.3)	0.9418
*sed*	2 (2.2)	0	0	1.0000
*see*	0	0	0	-
*seg*	18 (20.0)	2 (6.9)	0	0.0214
*seh*	23 (25.6)	6 (20.7)	6 (27.3)	0.8344
*sei*	19 (21.1)	6 (20.7)	1 (4.5)	0.1874
*sej*	2 (2.2)	0	0	1.0000
*sasX*	0	0	0	-

*lukS/F-PV*, gene encoding Panton-Valentine leukocidin

*tst*, gene encoding toxic shock syndrome toxin 1

*eta* and *etb*, gene encoding exfoliative toxin A and B

*sea*-*see* and *seg*-*sej*, gene encoding staphylococcal enterotoxins SEA-SEE and SEG-SEJ

*sasX*, gene encoding mobile genetic element

P-value, two-sided P-value calculated by the chi-square or Fisher’s exact test as appropriate.

### Molecular typing

**Fig 1:**
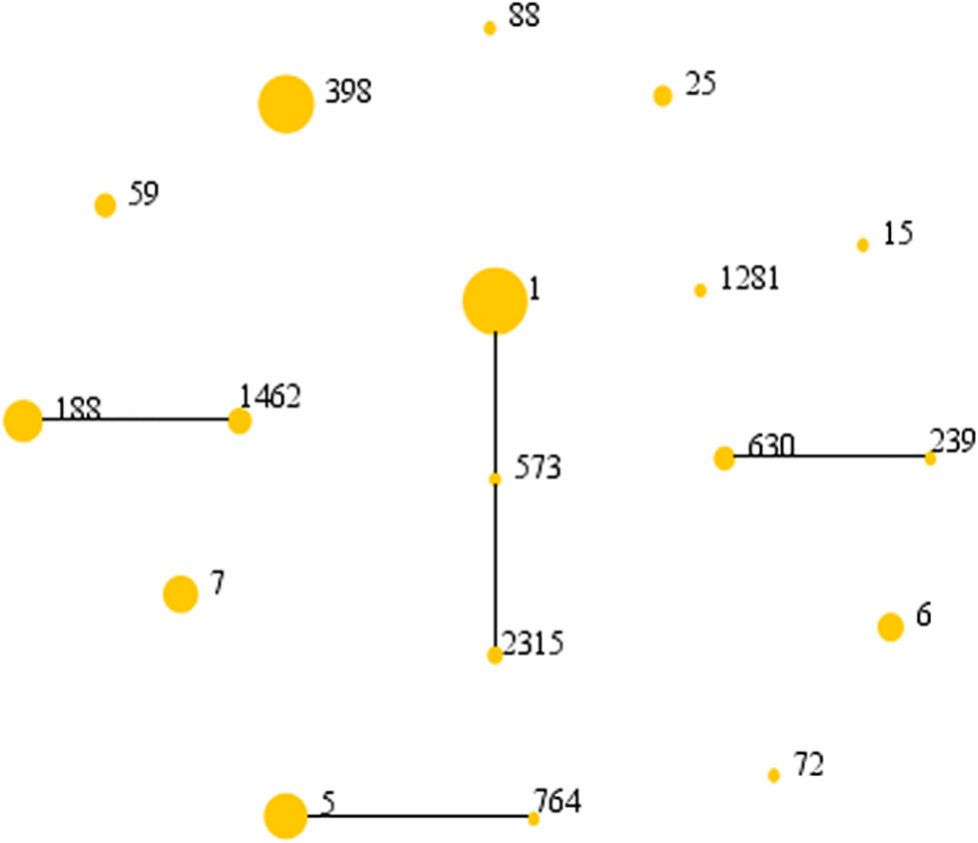
The diagram produced by eBURST with the stringent (default) group definition based on the MLST data of this study, representing the relationships of 141 *S*. *aureus* isolates. Each number represents an MLST ST and the area of each circle indicates the prevalence of the ST in the MLST data of this study.

Eighteen sequence types (STs) belonging to 13 clonal complexes (CCs) were identified among all 141 *S*. *aureus* isolates. As shown in Table 3 and Fig 1, CC1 (41/141, 29.1%) was the most common clone, followed by CC398 (28/141, 19.9%), CC188 (19/141, 13.5%) and CC5 (18/141, 12.8%). In the nasal carriers and axillary carriers, CC1 (26/90, 28.9%; 8/29, 27.6%, respectively) was the most common CC, and ST1 (24/90, 26.7%) was the most common ST in the nasal carriers while ST1 and ST398 (both 7/29, 24.1%) were the most common STs in the axillary carriers. The most common CC in the skin carriers was CC398 (8/22, 36.4%) and ST398 (8/22, 36.4%) was the most common ST. Two MRSA isolates from nasal carriers could not be sequence typed and *spa* typed (both negative for certain PCRs), and the SCC*mec* type of these two MRSA was V. In total, we detected 2 SCC*mec* type I (both from nasal carriers), 16 SCC*mec* type II (10 from nasal carriers; 6 from axillary carriers), 24 SCC*mec* type IV (14 from nasal carriers; 4 from axillary carriers; 6 from skin carriers), and 26 SCC*mec* type V isolates (15 from nasal carriers; 5 from axillary carriers; 6 from skin carriers). *spa* type was identified in 28 types in this study (S1 Table). t127 (31/141, 22.0%) was the most common *spa* type, followed by t14383 (18/141, 12.8%) and t002 (15/141, 10.6%). t127 (22/90, 24.4%) and t002 (6/29, 20.7%) was the most common *spa* type in the nasal carriers and axillary carriers respectively; t127 and t14383 (both 5/22, 22.7%) were the most common *spa* types in the skin carriers.

**Table 3:** Molecular characteristics of *S*. *aureus* isolated from residents in 7 NHs in Shanghai.

CC	No. of isolates	ST	SCC*mec* type	*spa* type (n)	Virulence genes (n)
1	41	1	I	t321(2)	*sea*(2), *sec*(2), *seh*(2)
		1	IV	t127(18), t14384(5)	*sea*(18), *sec*(22), *seh*(21)
		1		t127(13)	*sea*(8), *sec*(7), *seh*(10)
		2315		t11687(2)	*sec*(2), *seg*(2), *sei*(2)
		573		t14385(1)	*sec*(1), *seg*(1), *sei*(1)
5	18	5	II	t002(14), t688(1)	*tst*(8), *seb*(3), *sec*(7), *seg*(9), *sei*(14)
		764	II	t311(1)	*seb*(1), *seg*(1), *sei*(1)
		5		t002(1), t688(1)	*sec*(1), *sed*(2), *seg*(1),*sei*(2), *sej*(2)
6	6	6		t2467(3), t304(3)	*eta*(6), *sea*(6)
7	11	7		t091(9), t2616(2)	*sea*(1)
8	5	239	V	t037(1)	None
		630		t11041(4)	None
15	1	15		t803(1)	None
20	1	1281		t164(1)	*sei*(1)
25	3	25		t078(3)	*seb*(3), *seg*(3), *sei*(2)
59	4	59	IV	t3401(1)	*seb*(1)
		59	V	t437(2)	*lukS/F-PV* (2), *seb*(2)
		59		t441(1)	*seb*(1)
72	1	72		t148(1)	*tst*(1), *sec*(1), *seg*(1), *sei*(1)
88	1	88		t4333(1)	*lukS/F-PV* (1)
188	19	1462		t8275(5)	*seb*(4)
		188		t189(12), t2883(1), t3887(1)	*seb*(9)
398	28	398	V	t034(3), t14383(18)	*tst*(1)
		398		t034(7)	*tst*(1)
NT	2	NT	V	NT(2)	*seg*(2), *sei*(2)

CCs, clonal complexes; ST, sequence type by multi-locus sequence typing; SCC*mec*, Staphylococcal cassette chromosome *mec*; *spa*, Staphylococcus protein A gene; NT, not-typeable; None, no virulence gene detected.

Ten residents presented *S*. *aureus* in all three samples types and 12 residents presented *S*. *aureus* in two of the three samples (nasal & axillary, n = 8; nasal & skin, n = 1; axillary & skin, n = 3) as shown in Table 4. The clonal characteristics of *S*. *aureus* isolates from resident No. 43 and No. 44 were both different between nasal, axillary and skin samples, and the clones of *S*. *aureus* isolates from resident No.5 were different between nasal and axillary samples as well as resident No. 337 between axillary and skin samples. The clonal features of *S*. *aureus* isolates from the different samples of the remaining 18 residents were found to be the same.

**Table 4:** Characterization of 22 residents that each one isolated *S*. *aureus* from different samples.

Resident No.	Age/Gender	Sample type	Clone	Institution
1	73/Female	Nasal, Axillary, Skin	ST398-t034	NH1
19	84/Female	Nasal, Axillary, Skin	ST398-SCC*mec*V-t14383	NH1
39	85/Female	Nasal, Axillary, Skin	ST398-SCC*mec*V-t14383	NH1
43	76/Male	Nasal	ST1-t127	NH1
		Axillary, Skin	ST188-t189	
44	81/Male	Nasal	ST7-t091	NH1
		Axillary	ST1-SCC*mec*IV-t14384	
		Skin	ST1-t127	
65	85/Male	Nasal, Axillary, Skin	ST398-SCC*mec*V-t14383	NH1
105	91/Female	Nasal, Axillary, Skin	ST1462-t8275	NH2
207	86/Female	Nasal, Axillary, Skin	ST398-SCC*mec*V-t034	NH2
350	91/Female	Nasal, Axillary, Skin	ST189-t189	NH5
492	85/Female	Nasal, Axillary, Skin	ST1-SCC*mec*IV-t127	NH7
5	85/Female	Nasal	ST7-t091	NH1
		Axillary	ST398-t034	
16	58/Male	Nasal, Axillary	ST6-t034	NH1
221	80/Female	Nasal, Axillary	ST1-t127	NH2
301	86/Female	Nasal, Axillary	ST5-SCC*mec* II-t002	NH4
344	78/Female	Nasal, Axillary	ST5-SCC*mec* II-t002	NH5
361	85/Female	Nasal, Axillary	ST188-t189	NH5
371	81/Female	Nasal, Axillary	ST2315-t11687	NH5
478	86/Female	Nasal, Axillary	ST59-SCC*mec*V-t437	NH7
64	83/Female	Nasal, Skin	ST398-SCC*mec*V-t14383	NH1
62	93/Female	Axillary, Skin	ST1-SCC*mec*IV-t127	NH1
144	87/Female	Axillary, Skin	ST6-t2467	NH2
337	81/Female	Axillary	ST5-SCC*mec* II-t002	NH5
		Skin	ST1-SCC*mec*IV-t127	

Clone, ST-SCC*mec*-*spa*-type; ST, sequence type by multi-locus sequence typing; SCC*mec*, Staphylococcal cassette chromosome *mec*; *spa*, Staphylococcus protein A gene.

## Discussion

To our best knowledge, there is no published epidemiological data of *S*. *aureus* colonization in NH residents in China. This survey conducted in Shanghai has investigated the prevalence of *S*. *aureus* carriers among 491 elderly residents from 7 NHs in Shanghai. The overall prevalence rates of *S*. *aureus* and MRSA colonization in this study are 22.2% and 10.6% respectively. As reported in other countries, the prevalence rate of MRSA in the resident population varies: 23.3% in Northern Ireland[3], 7.1% in Croatia[17], 4.7% in England[7], 10.1% in Ireland[18], 7.6% in Germany[19], 19.9% in Belgium[1], and 16% and 26% in the United States [2]. Another report in Italy has discovered that the MRSA strains isolated from long-term care facilities were resistant to ciprofloxacin and the majority was susceptible to most other non-betalactam antibiotics [20]. The high resistance rates of MRSA include 61.8% to ciprofloxacin, 70.6% to kanamycin, 66.2% to tobramycin, and 66.2% to erythromycin. Antibiotic resistance has a major impact on mortality among elderly patients, and high rate of MRSA colonization was discovered in this study. The result reveals there is a lot to be desired in terms of improving infection control measures among different health care settings. At the same time, using more appropriate antibiotics therapy in order for the quality of care and overall outcomes also works.

CC1 was found to be the most common CC within all the isolates of *S*. *aureus* studied (41/141, 29.1%), followed by CC398 (28/141, 19.9%), CC188 (19/141, 13.5%) and CC5 (18/141, 12.8%). And CC1 (26/68, 38.2%), CC398 (21/68, 30.9%) and CC5 (15/68, 22.1%) were the most common CCs within MRSA isolates. ST5-t002/t242 has been previously reported as the dominant MRSA clone in NHs in a study conducted in California, United States [2], and t002 was also the common *spa* type that we found in our study. Nevertheless, ST8 which was found as the predominant ST in NHs in both Belgium and the Unites States [1, 2] was not discovered at all in our current study, and CC8 (ST239, ST630) was found in only 5 isolates. CC8 (ST8, ST239) was predominant in hospitals across all continents, and ST239 was a common epidemic ST in many *S*. *aureus* infections including bloodstream infection and surgical site infections in China[13, 15]. ST1 and ST5 were both found as major clones in infected infants in a neonatal intensive care unit in Portugal[21], perhaps also playing a role in *S*. *aureus* reservoir and transmission routes. Another report has recognized CC5 as another dominant clone in hospitals in Shenyang, China[22]. ST398 has been reported causing an outbreak of MRSA in a Dutch Nursing Home between 2010 and 2011[23]. CC398 was also recently reported as a livestock-associated clone in skin and soft-tissue infections (SSTIs) in China[24, 25] and the *spa* type t034 (CC398) which was found in 3 MRSA and 7 MSSA was reported as a dominant type in pigs and pig farmers in Canada[26]. ST188 was the common genotype of methicillin-susceptible *S*. *aureus* (MSSA) found in invasive community-acquired *S*. *aureus* infection in Chinese children[27].

Sixty-eight MRSA strains were observed in 52 residents. Among these 52 residents, 5 residents were tested positive for MRSA in all three sample types and 6 residents were tested positive for MRSA in two sample types. Twelve of the 52 residents had a history of surgical procedure; 9 residents were being administered with catheters or other percutaneous medical devices; and 13 residents were undergoing catheters or indwelling percutaneous medical devices in addition having a history of surgery. In this study, SCC*mec* type IV and V which usually associated with community-associated MRSA (CA-MRSA) were detected in 50 isolates (73.5%). Assessing the extent to which traditionally CA-MRSA has penetrated the nursing home reservoir is of key interest. High prevalence of CA-MRSA has been reported here and CA-MRSA penetration into nursing homes certainly may require additional infection control measures [28]. Livestock-associated MRSA (LA-MRSA) CC398 was found in 21 isolates, and the SCC*mec* type of all was V. Prophages of integrase group 3 which contains the immune evasion gene cluster (IEC) are usually carried by *S*. *aureus* adapted to humans that integrate into the β-hemolysin gene[29]. The IEC genes (*chp*, *sak*, *scn*) were found in all the 21 LA-MRSA isolates in this study, thus it might imply the LA-MRSA isolates were acquired from an ancestral human subpopulation. In particular, 17 of the 21 LA-MRSA isolates were sourced from 10 residents in NH1 and all belong to ST398-SCC*mec*V-t14383. Three isolates (ST398-SCC*mec*V-t034) were from the same resident in NH2 and the remaining one (ST398-SCC*mec*V-t14383) was from NH7.

Three samples (nasal, axillary and skin swabs) were collected from one resident at the same time in our study. It is important to note that 4 residents (resident No. 43, No. 44, No.5 and No. 337 in Table 4) were found to have *S*. *aureus* colonize in at least two samples, and the molecular characteristics of the *S*. *aureus* isolates from different samples among each resident were different as shown in Table 4. This may imply that one person could be colonized by different sourced *S*. *aureus* in different sites of the body, and samples from different sites should be collected when conducting the *S*. *aureus* screening and evaluating the elimination of *S*. *aureus* colonization.

*lukS/F-PV* was discovered in only three isolates, two of which were ST59 MRSA from the same resident (nasal and axillary swabs) and the remaining one being ST88 MSSA (from nasal swab), which happen to be the common *lukS/F-PV*-positive clones in other *S*. *aureus* infections in China[13, 30]. The gene *tst* encoding toxic shock syndrome toxin 1 (TSST-1) was found in 11 isolates (8 ST5 MRSA, 1 ST398 MRSA, 1 ST398 MSSA and 1 ST72 MSSA). It is notable that the TSST-1 ST5 clone has been described in France and China before [13, 15, 31]. The enterotoxin genes *seg* and *sei* are typically associated to the *egc* cluster and variants [32]. However we found 7 isolates *sei*-positive but *seg*-negtive, which implies the possibility of a new *egc* variant. This is an area of work which can be explored in further studies. Six (5 MRSA and 1 MSSA) of these 7 isolates belong to ST5 and the other one (MSSA) was ST1281.

Nursing homes are known as specialized nursing facilities for the elderly, and are also found to be rather significant reservoirs for *S*. *aureus*. The limitation in our study was that health care workers (HCWs) and staff in NHs were not enrolled in the study design and we lack the information regarding any associated *S*. *aureus* colonization of these HCWs and staff. Unfortunately, the infection control education and training program has not proved to reduce MRSA prevalence in nursing homes[33, 34]. Other measures should be considered and specifically designed to reduce the prevalence of MRSA colonization among residents and staff, and more intense and focused education and training are needed. One modeling study predicts that MRSA may persist without strict screening and decolonization of colonized individuals at admission. The possible control strategies include decolonizing colonized residents, improving hand hygiene in both residents and HCWs, reducing the duration of contamination of HCWs and decreasing the resident to staff ratio [35]. There are also ways to potentially reduce resistance to antimicrobial agents in NHs including increase in number of staff, increase the amount of hand-washing basins, and use of antimicrobial soap [36]. It was suggested that modifying cleaning practices may reduce both MRSA environmental contamination and patient burden among NHs[37], and that the best practices of infection control and MRSA surveillance information should in fact be shared among hospitals and nursing homes[38]. Another modeling study suggests that models have the ability to accurately predict the probability of nasal carriage of MRSA as well as positive predictive values of various rapid diagnostic tests when given a set of resident parameters[39]. Last but not least, empiric antibiotic treatment, infection control measures and screening method may be applied to rectify colonization of *S*. *aureus* and MRSA. Increased convenience and feasibility may also be realized with these control strategies for *S*. *aureus* and MRSA colonization.

## Supporting Information

S1 Table Information and molecular characteristics of 141 *S*. *aureus* isolates among residents of seven nursing homes in Shanghai. (XLSX)
